# Fiber Reinforcement of Soft Spider Silk Hydrogels

**DOI:** 10.1002/marc.202500475

**Published:** 2025-09-16

**Authors:** Christina Heinritz, Thomas Scheibel

**Affiliations:** ^1^ Chair of Biomaterials Faculty of Engineering Science University of Bayreuth Bayreuth Germany; ^2^ Bayreuth Center for Colloids and Interfaces (BZKG) Bavarian Polymer Institute (BPI) Bayreuth Center for Molecular Biosciences (BZMB) Bayreuth Center for Material Science (BayMAT) University of Bayreuth Bayreuth Germany; ^3^ Faculty of Medicine University of Würzburg Würzburg Germany

**Keywords:** biofabrication, composites, fibers, fibrils, self‐assembly

## Abstract

Recombinant spider silk‐based biomaterials show high application potential due to their biocompatibility, biodegradability, and low immunogenicity. Self‐assembly of monomeric proteins into nanofibrils is necessary toward hydrogel formation and yields a dense physically entangled network, in which cells show high viability but so far low proliferative activity. To facilitate enhanced cell activity and growth, in this study low‐concentration spider silk hydrogels were fabricated, resulting in higher cell proliferation but suffering from poor mechanical stability. Thus, electrospun fiber meshes also made from spider silk proteins were integrated into the soft hydrogels using a layer‐by‐layer approach. The composite structure significantly improved the mechanical properties and shape fidelity, including an increase in Young's modulus by an order of magnitude, while preserving the hydrogels’ biocompatibility. This work provides a promising strategy for developing mechanically reinforced, cell‐friendly spider silk‐based hydrogels suitable for soft tissue engineering applications.

## Graphical abstract

Along the development of matrices for biomedical applications, precise tuning of the biomaterial to interact with specific cell types/tissues is essential. The fabrication of fiber mesh‐reinforced soft hydrogels made of recombinant spider silk proteins allows for the adjustment of mechanical properties and hydrogel porosity, leading to improved cell response.

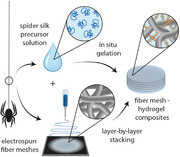

## 1 Introduction

Biofabrication aims to develop in vitro models for e.g. analyzing diseases, drug testing, but also the repair or regeneration of tissue defects using a combination of cells, signaling molecules and matrices [1, 2]. The choice of the matrix is of great importance and should be carefully designed for and adjusted to the needs of the respective cells/tissue. In natural tissues, the extracellular matrix (ECM) is a 3D network providing biochemical (ligands, bioactive factors) as well as biophysical (charge, degradability, surface topography, and viscoelasticity) cues to the surrounding cells [3]. From a structural point of view, hydrogels show a porosity and viscoelasticity mimicking some features of native ECM and are among the most frequently used scaffolds in biofabrication [4]. Key properties to be considered for such hydrogels are porosity, mechanical strength/stiffness, swelling behavior, adhesion to the tissue substrate, biodegradability and biocompatibility [5].

The reaction of cells to a hydrogel scaffold is significantly influenced by the mechanical properties of the underlying material [1]. Cells detect the matrix stiffness and convert the mechanical signals into physiological responses like cell growth and differentiation. For example, hematopoietic stem cells show less proliferation and increased quiescence in presence of stiffer hydrogels [6], and mesenchymal stem cells react with distinct lineage specification to differences of matrix stiffness [7]. Further, the mechanics of the cell environment influence the vascular development [8], and aberrant alterations are linked to various pathological conditions including cancer development and progression [9]. In general, the human body encompasses a striking range of stiffness depending on the tissue of interest: from soft native brain tissue (modulus *E*_brain_ ∼1–3 kPa) to most abdominal organs, skin and muscle (e.g. *E*_liver_ ∼4.0–6.5 kPa, *E*_skeletal muscle_ ∼5–170 kPa) to bones with the highest stiffness in the GPa range (*E*_bone_ ∼1–20 GPa) [10].

In case of hydrogels, the stiffness can be controlled by the polymer volume fraction increasing the physical entanglement and chemical crosslinking degree. However, increasing these parameters results in a denser network structure and yields a decreased pore size and porosity as trade‐off. Based thereon, the permeability of the hydrogel is reduced [11], affecting e.g. the exchange of oxygen and nutrients as well as cell migration and infiltration of the matrix [5]. To balance sufficient pore size and adequate mechanical properties is challenging [12], thus, an increasing number of studies has addressed the mechanical reinforcement of soft hydrogels while maintaining high porosity. Some strategies, for example, included the application of more than one material to create interpenetrating networks [13], the use of sacrificial materials soluble in water [12] or organic solvents [14], or filler materials like fibers, fiber fragments, or particles [15, 16, 17].

**FIGURE 1:**
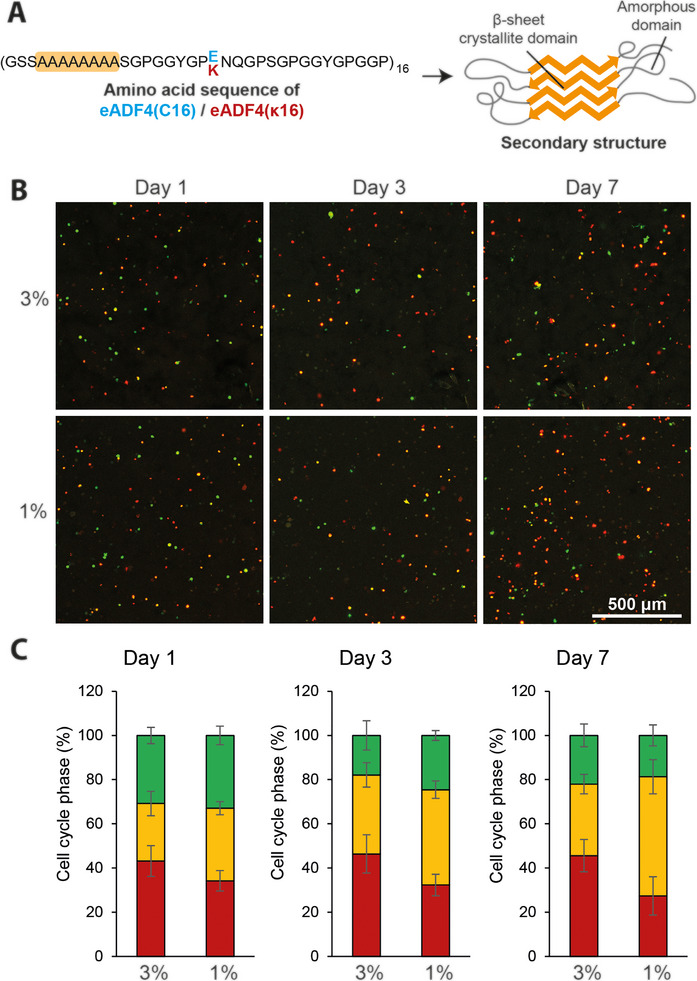
Cultivation of fibroblast NIH/3T3 FUCCI proliferation‐reporter cells in 3% (w/v) and 1% (w/v) eADF4(C16)‐RGD hydrogels, respectively. (A) Schematic representation of the primary and secondary structure of the recombinant spider silk proteins eADF4(C16)/eADF4(κ16). The poly‐alanine blocks form β‐sheet‐rich crystalline regions, whereas the GPGGX motifs yield amorphous structures. (B) The reporter cells were incubated in eADF4(C16)‐RGD hydrogels at concentrations of 3% / 1% (w/v) as indicated. The FUCCI system of the reporter cells (see materials and methods) enabled the observation of cell cycle progression from cell cycle phase G1 (red) to S/G2/M phases (green) at day 1, 3, or 7 as indicated. The G1/S transition appears yellow due to the presence of both fluorescent proteins. Representative confocal images display a maximum projection up to a depth of 100 µm. Scale bar = 500 µm. (C) Quantitative analysis of reporter cells in the proliferative state (S/G2/M phases, green, and G1/S transition phase, yellow) and in G1 phase (red) with respect to the total number of cell nuclei.

In this context, hydrogels made of spider silk‐based proteins have been used for various applications in tissue engineering [18]. Biotechnological production of recombinant spider silk proteins allows for the production of sufficient amounts of proteins and enables the modification of naturally derived protein sequences. We have published substantial work on the engineered fibroin 4 from *Araneus diadematus* (eADF4(C16)) comprising 16 repeats of a core sequence called C‐module [19]. The negatively charged eADF4(C16) has been further genetically engineered to substitute all glutamic acid residues to lysine ones yielding the positively charged eADF4(κ16) [20]. Both spider silk variants were found to fibrillize with their poly‐alanine sequence motifs forming antiparallel β‐sheets and the remaining glycine‐rich regions building an amorphous matrix (Figure 1A) [21]. Self‐assembly yielded fibrils with diameters of 3–8 nanometers [22, 23], which at higher protein concentrations formed networks by physical entanglement resulting in a hydrogel. Fibril formation follows a nucleation mechanism with a slow initial oligomerization step yielding a nucleus and a fast fibril‐elongation step [24]. Importantly, assembly kinetics can be influenced in the presence of kosmotropic ions such as phosphate [25], by shear stress [26], by increased protein concentration, and at increased temperature [27]. Recombinant spider silk hydrogels have been used for the encapsulation and cultivation of cells, especially when genetically modified protein variants were used comprising the integrin recognition sequence RGD [28, 29]. 3% (w/v) spider silk hydrogels have been well characterized regarding self‐assembly, secondary structure and cell compatibility and have been also tested e.g. concerning vascularisation in vivo, where hydrogels made of the protein variant eADF4(C16)‐RGD supported the sprouting and growth of blood vessels [29, 30, 31, 32]. Due to their slow biodegradation found in vitro and in vivo [32, 33, 34, 35, 36], as well as their inherent microbe‐repellent properties [37], these hydrogels are a promising stable matrix for tissue engineering.

Further, due to the shear‐thinning behavior of spider silk hydrogels, they can be used as bioinks, with embedded cells showing a high viability after dispense plotting. However, so far in these printed structures cells were lacking proliferative activity [28]. A recent study demonstrated that soft eADF4(C16) bioinks at lower protein concentrations mechanically enforced with collagen‐I coated polycaprolactone (PCL) fiber fragments led to increased proliferation rates post‐printing, indicating an improved microenvironment for cells [15].

In this study, we intended to combine low‐concentrated soft spider silk hydrogels with electrospun spider silk fibers to improve the performance of such scaffolds for biofabrication. Recombinant spider silk proteins can be easily processed into continuous fibers using electrospinning [38]. The resulting fibers in the submicro‐ to low micrometer range resemble some morphological characteristics of the natural ECM [39, 40]. Correspondingly, electrospun eADF4(C16) meshes were previously found to support cell adhesion and proliferation by topographical cues [41]. Here, a combination of the two morphologies, electrospun fibers and hydrogels were fabricated, providing a more natural‐like environment for cells and enabling manipulation of the mechanical properties of the newly gained bulk scaffold.

Amongst various approaches that can be pursued to produce structurally supported composite scaffolds, stacking of individually manufactured fiber meshes and hydrogels (laminating approach) was chosen in this study, since it is the simplest yet most effective method [42, 43].

## 2 Results and Discussion

### 2.1 Cell Growth in Spider Silk Hydrogels with Low Viscosity

**FIGURE 2:**
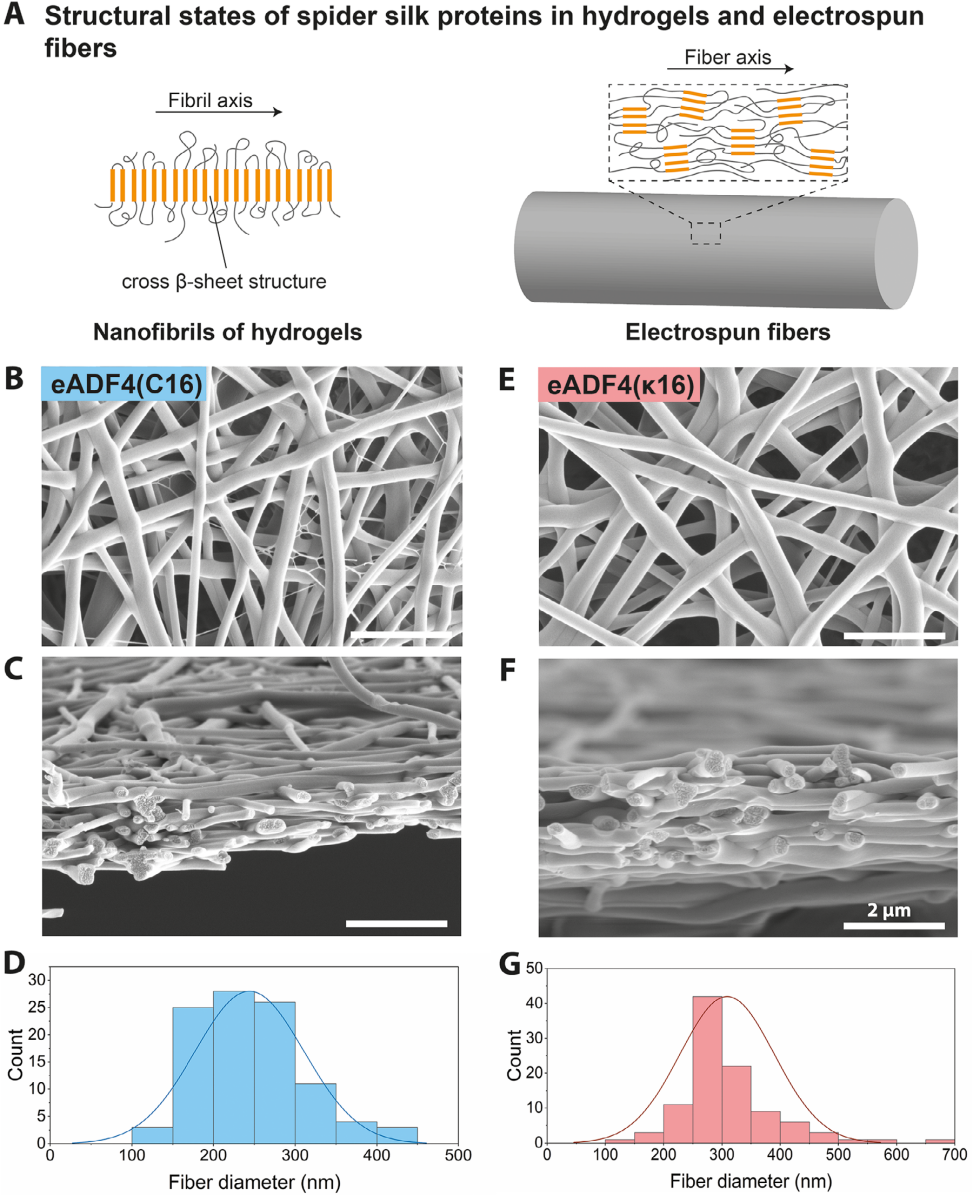
(A) Self‐assembly of the spider silk proteins into 3–8 nm wide fibrils yields hydrogels with cross β‐sheet structures perpendicular to the fibril axis. Electrospinning of soluble spider silk proteins yields fibers with diameters in the range of 100–1000 nm. Therein, β‐crystals are embedded in an amorphous matrix and align presumably along the fiber axis. (B, C) Representative SEM images of electrospun fiber meshes using spinning dopes with 12.5% (w/v) eADF4(C16) and (E, F) 15% (w/v) eADF4(κ16) in hexafluoroisopropanol (HFIP) after post‐treatment with methanol vapor, showing the surface and the cross‐section of a mesh (scale bar = 2 µm). (D) Diameter distribution of eADF4(C16) fibers with a mean diameter of 244 ± 67 nm and (G) of eADF4(κ16) fibers with a mean diameter of 309 ± 81 nm.

Since proliferative activity of cells is limited in mechanically stable, highly viscous 3% (w/v) spider silk hydrogels, cell proliferation was tested in 1% (w/v) hydrogels, which is at the lower limit of concentration at which such hydrogels can self‐assemble [27, 44]. Murine NIH/3T3 fibroblasts were used for these tests stably producing the fluorescent ubiquitination‐based cell cycle indicator (FUCCI), including two fluorescently labelled proteins that are reciprocally active during the cell cycle, in particular the fusion proteins kusabira‐orange‐cdt1 (red fluorescence) in the G1 phase of the cell cycle and azami green‐geminin (green fluorescence) in the S/G2/M phase [45]. Thus, the color of the nuclei indicates the phase of the cell cycle, which can be visualized and quantified via fluorescence microscopy [46]. Direct comparison of the two hydrogel concentrations/viscosities showed higher proliferative activity inside the less viscous, lower concentrated hydrogel represented by the higher fraction of green (S/G2/M) and yellow nuclei (G1/S transition) (Figure 1). With time, the difference became even more pronounced. This finding suggested that 1% spider silk hydrogels are more favorable for cell proliferation than 3% hydrogels. However, the 1% hydrogels were very soft, had low shape fidelity, and were tricky to handle. To overcome these three drawbacks, composite hydrogels were assembled with integrated fiber meshes for mechanical reinforcement without increasing the polymer content of the hydrogel fraction (Figure 2A).

### 2.2 Fabrication of Spider Silk Fiber Mesh—Hydrogel Composites (fmhc)

A 12.5% (w/v) solution of eADF4(C16) dissolved in 1,1,1,3,3,3‐hexafluoro‐2‐propanol (HFIP) was processed using electrospinning as previously described [38]. yielding randomly oriented fibers with a mean diameter of 244 ± 67 nm (Figure 2B,D). By transfer of the non‐woven meshes to a frame modified with sticky tape, free‐standing meshes were obtained for a simplified handling (Figure S1), which had a cross‐sectional thickness of a few microns (Figure 2C). To study if electrostatic interactions between oppositely charged fibers and the self‐assembling hydrogel can enhance the mechanical properties of the composite, electrospun meshes of the positively charged recombinant spider silk protein eADF4(κ16) were also produced showing a mean fiber diameter of 309 ± 81 nm and a similar cross‐sectional thickness as the ones made from eADF4(C16) (Figure 2E–G). All electrospun recombinant spider silk fiber meshes were post‐treated with methanol to increase the content of β‐sheet crystals and, thus, yield water resistance [47]. Analysis of the fiber meshes using Fourier transform infrared (FTIR) spectroscopy before and after methanol post‐treatment confirmed the structural conversion of both electrospun spider silk protein variants (Figure S2).

**FIGURE 3:**
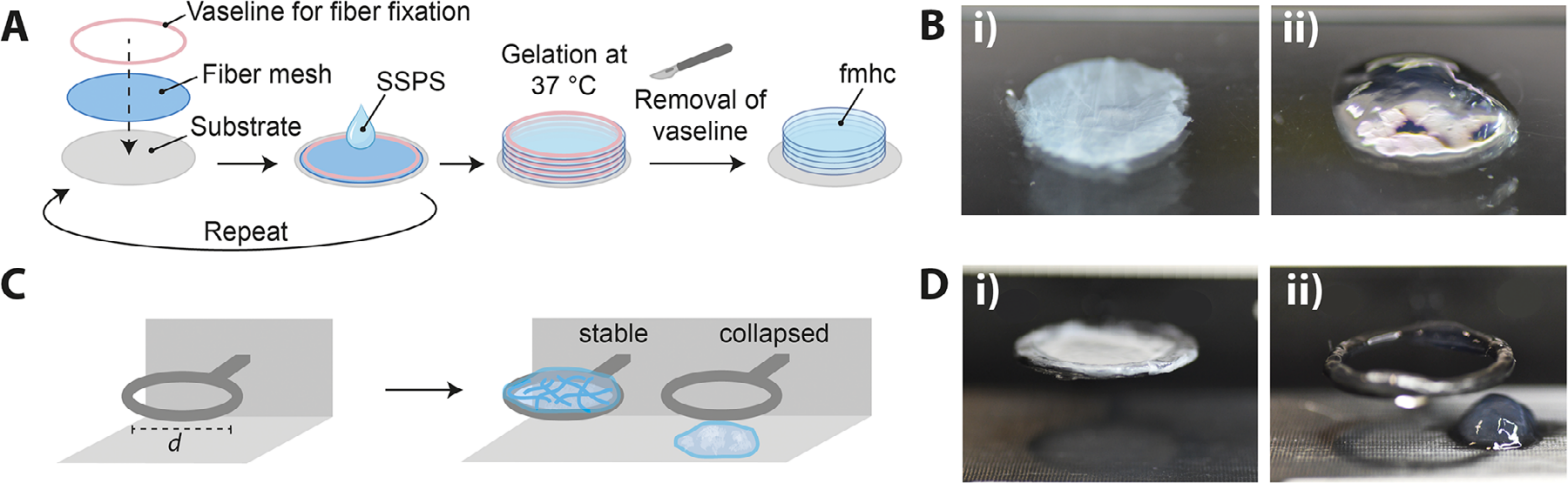
(A) Schematic drawing showing the workflow of the assembly process to produce multi‐layered laminated fiber mesh–hydrogel composites (fmhc) using electrospun spider silk fiber meshes and spider silk precursor solutions (SSPS). (B) Photographs of the resulting fmhc scaffold (i) compared to the soft hydrogel without fiber enforcement (ii). (C,D) To test the improvement of the macroscopic stability upon incorporating fiber meshes, PLA (polylactic acid) rings were produced as testing devices with various diameters *d*. The fmhc was able to bridge the inner hole of a ring with a diameter of 15 mm (i), whereas the hydrogel without fiber mesh support collapsed (ii).

Electrospun spider silk fiber meshes and 1% spider silk precursor solutions (SSPS) were combined in a layer‐by‐layer approach with in situ gelation of the SSPS (Figure 3A). For the assembly of the multi‐layered composite, Vaseline was used as sealant, fixing the fiber layers and at the same time sealing the structure against leakage when applying the SSPS. The solution was applied between non‐woven layers followed by gelation at 37°C for 7 days after fabrication of the multilayer composite. The resulting 1% (w/v) eADF4(C16) hydrogel with eADF4(C16) fibers was termed fmhc_CC_ and the 1% (w/v) eADF4(C16) hydrogel with eADF4(κ16) fibers was termed fmhc_CK_. The weight percentage of fibers in the fmhc was roughly estimated to be below 1%, however, the composites appeared less transparent compared to the hydrogel without fiber mesh support due to the light dispersion of the fibers (Figure 3B).

However, an increased stability was already visible on a macroscopic scale, demonstrated by a newly designed experimental setup to test the ability of the hydrogels to stably bridge circular holes with different diameters (Figure 3C). While the hydrogel failed and collapsed at a ring size with an inner diameter of 15 mm, the fmhc was able to bridge such ring, pointing out an increased stability and mechanical support provided by the fiber meshes (Figure 3D).

### 2.3 Morphology of the fmhc

**FIGURE 4:**
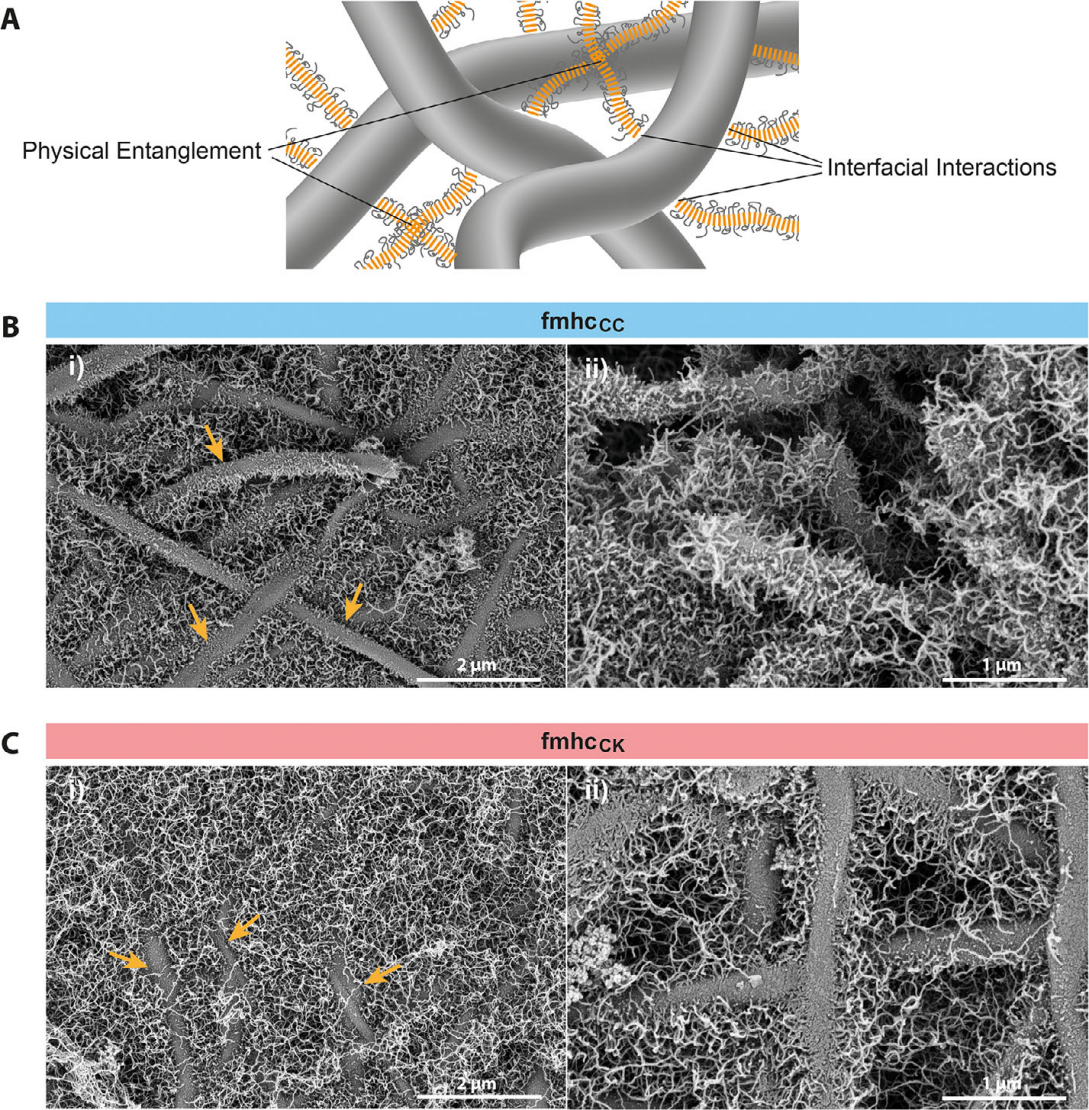
Fmhc show fibers (some highlighted with arrows) embedded in a fibril network employing physical entanglement and interfacial interactions, schematically displayed in (A). Representative SEM images of 1% (w/v) eADF4(C16) hydrogels reinforced with (B) eADF4(C16) or (C) eADF4(κ16) fiber meshes. At higher magnification (ii), a brush‐like fibril anchorage is visible on the fiber surfaces. The short fibril length in ii) is presumably an artefact resulting from fibril breakage during sample preparation to enable visualization of the layer interfaces and is not seen in i). Scale bars (i) 2 µm, (ii) 1 µm.

On a microscopic scale, the morphology of fmhc was characterized using SEM. For better preservation of the ultrastructure, scaffolds were critical‐point dried after chemical fixation and dehydration using an ethanol series. SEM images showed a network of (self‐assembled) fibrillar structures in the nanometer range, embedded within the mesh of eletrospun fibers and filling the gaps of the porous meshes (Figure 4B‐i,C‐i). Strikingly, the eADF4(C16) nanofibrils seemed to extend from the surface of eADF4(C16) electrospun fibers leading to a brush‐like morphology (Figure 4B‐ii). This suggests that the fiber surface can act as secondary nucleation site as published previously [48], triggering the self‐assembly of the spider silk proteins into fibrils in alignment with other previously published fibril assembly studies on the surface of particles [24] and coatings [49] made of eADF4(C16). The fibril growth from the fiber surface is somewhat resembling a “grafting‐from” approach where polymerization from a solid surface yields a brush‐like polymer structure [50]. SEM images of electrospun eADF4(κ16) fiber meshes embedded in the hydrogel showed surprisingly similar fibril anchorage on the fiber surfaces (Figure 4C‐ii). Therefore, stable integration and bonding of the non‐woven layers (independent of the fiber's surface net charge) with the hydrogel network is assumed, which should have considerable influences on the mechanics of laminated composites (Figure 4A) [51].

### 2.4 Mechanical Testing of fmhc

**FIGURE 5:**
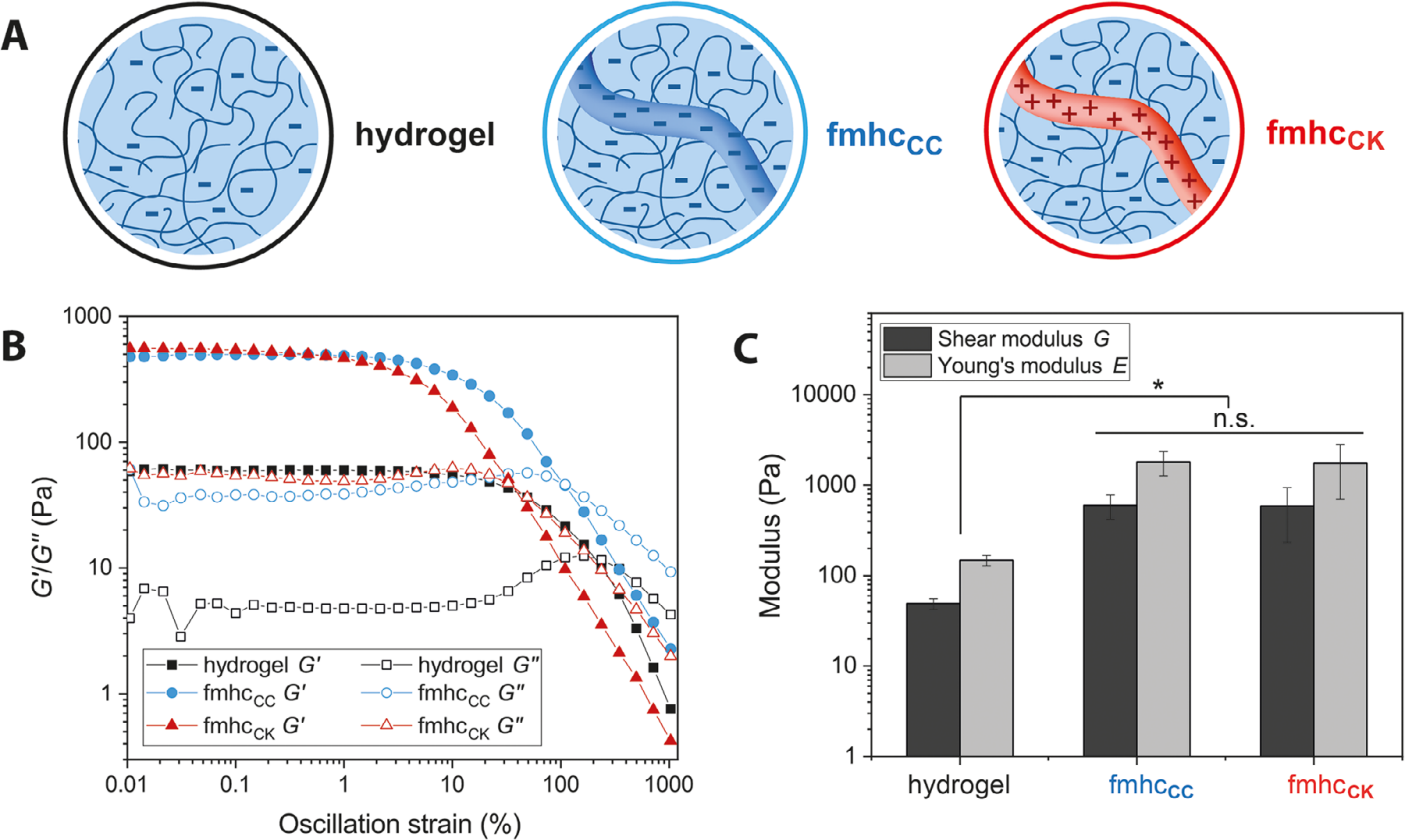
(A) Analyzed samples: 1% (w/v) eADF4(C16) hydrogels (black, hydrogel), 1% (w/v) eADF4(C16) hydrogels with integrated multilayers of electrospun eADF4(C16) fibers (blue, fmhc_CC_) or eADF4(κ16) fibers (red, fmhc_CK_). (B) Exemplary rheological curves of the amplitude sweep (storage modulus *G’* filled symbols, loss modulus *G″* empty symbols). (C) Comparison of the shear and Young's moduli of the linear viscoelastic region reveals an increase of more than a magnitude for the multilayer fmhc. Data: mean ± SD. Statistics: *p < 0.05, n.s. not significant.

Rheological measurements can be used to obtain the storage modulus of a hydrogel in response to radial strain within its linear viscoelastic range in parallel plate rheology experiments. The results thereof are often treated as equivalent estimate for the shear modulus [52, 53]. Figure 5B shows curves of oscillatory amplitude sweep measurements of spider silk hydrogels with G’ > G″ indicating gel‐like properties. The pure 1% (w/v) eADF4(C16) hydrogel without fiber enforcement showed a linear viscoelastic region (LVE) up to ∼15% strain, resulting in destruction of the hydrogel network at higher strains. The addition of fiber meshes reduced the linear region. However, storage and loss moduli were an order of magnitude higher in fmhc. The Young's modulus of 0.15 kPa of the hydrogel increased to 1.81 and 1.76 kPa in case of fmhc, respectively, with the difference between fmhc_CC_ and fmhc_CK_ not being statistically significant (Figure 5C). Along the development of fmhc, it is assumed that the integration of a ductile fiber network into a hydrogel matrix increases the stiffness and fracture toughness of the hydrogel and improves energy absorption and prevents crack propagation [54, 55]. In accordance, the integration of multiple layers of fiber meshes led to a decreased deformation within the shearing environment and, thus, to an increased stiffness of the hydrogels [56]. Interestingly, since both used fiber meshes were similar except the surface net charge of the fibers (based on the underlying proteins) (Figure 5A), we concluded that electrostatic interactions did not play a significant role in the mechanical reinforcement of the hydrogels. Rather the interfacial fibril assembly nucleation presumably initiated by the fiber surfaces ensured sufficient stress transfer from the soft hydrogel to the stiffer fibers. In summary, the introduction of fiber meshes increased the bulk modulus of the composite scaffold without altering the formulation of the hydrogel fraction and is within the range of soft tissues, making it a promising scaffold for e.g. neural tissue engineering.

### 2.5 Cell Compatibility of the fmhc

To determine the effect of fiber‐reinforcement on the proliferation of cells, cells were seeded on top of the fmhc, and proliferation was compared to that on 1% (w/v) control hydrogels. Only one layer of electrospun non‐woven meshes was used, facilitating microscopic analysis, and eADF4(C16)‐RGD was used for the hydrogel fraction to achieve cell attachment as reported previously [57]. Cultivated proliferation‐reporter cells showed similar ratios of cell cycle phases in absence or presence of electrospun fiber meshes (Figure S3A). Examination of the morphology of cells after 24 h of cultivation on top of the scaffolds showed spread cells expressing stress fibers on all matrices, indicating cytocompatibility and no negative effect of the embedded fiber meshes on the cells’ behavior (Figure S3B,C).

## 3 Conclusion

In this study, we successfully enhanced the mechanical performance of low viscosity recombinant spider silk‐based hydrogels by incorporating electrospun spider silk fiber meshes using a layer‐by‐layer approach. The resulting composite material showed significantly improved structural integrity. SEM images indicated apparent growth of fibrils from electrospun fiber surfaces, which presumably act as secondary nucleation sites for the self‐assembly of soluble spider silk proteins into β‐sheet rich fibrils, resembling “grafting‐from” approaches known in the field of polymers. We hypothesize that the assembly starts on the fiber surfaces leading to an anchorage of fibers in the hydrogel network, and thus, reducing slippage and delamination of individual layers, typically critical in laminated constructs.

Importantly, the low concentration of hydrogels supported higher proliferative activity of cells on their surface, making these fmhc suitable for biomedical applications and tissue engineering [58]. Overall, our findings offer a promising strategy for the development of advanced composite biomaterials with tunable mechanical properties and enhanced functionality.

## 4 Experimental Section

### 4.1 Preparation of Recombinant Spider Silk Hydrogel Precursor Solutions (SSPS)

Recombinant spider silk proteins eADF4(C16), eADF4(C16)‐RGD and eADF4(κ16) were produced and purified as published previously [19, 20]. Lyophilized proteins were dissolved in 6 M guanidinium thiocyanate (Carl Roth GmbH & Co. KG, Karlsruhe, Germany) at 20 mg mL^−1^ for 1 h at room temperature to solubilize aggregated proteins. Dialysis against 10 mm Tris/HCl pH 7.5 was conducted using dialysis membranes with a molecular weight cut‐off of 6–8 kDa with 3 buffer exchanges overnight at room temperature. The resulting aqueous protein solutions were centrifuged after dialysis at 17 000 x *g*, 4°C for 10 min. The final protein concentration was determined photometrically at 280 nm and adjusted to 10 mg mL^−1^ using the same buffer. As an advantage over higher concentrated silk solutions, no osmotic pressure‐driven removal of water via dialysis against a polyethylene glycol solution was necessary to reach the final protein concentration, facilitating the hydrogel preparation process. SSPS were stored at 4°C up to 14 days.

### 4.2 Electrospinning of Recombinant Spider Silk Proteins

Lyophilized eADF4(C16) (12.5% w/v) and eADF4(κ16) (15% w/v) were dissolved in 1,1,1,3,3,3‐hexafluoro‐2‐propanol (HFIP, abcr GmbH, DE) and filled in 1 ml syringes with a 21G blunt end needle (Sterican, B Braun SE, DE). Electrospinning was performed using a custom‐built setup with 18 cm distance between the needle and the plate collector at a potential difference of 25 kV, a feeding rate of 4 µL/min, and a controlled relative humidity of 15–30%. For easier removal from the substrate, fibers were spun for 5 min on black paper, which was put on top of the metal plate collector together with a circular shielding for focusing. After spinning, fiber meshes were transferred onto frames (custom‐3D‐printed from polylactic acid with a 4.5 cm diameter wide hole in the center) using double sided tape (Figure S1). Free‐standing meshes fixed in this way were then post‐treated in a 100% methanol (VWR, Darmstadt, Germany) vapor atmosphere overnight to induce crystalline structures and render the samples water insoluble.

### 4.3 Assembly of A Multilayered Fiber Mesh–Hydrogel Composite (fmhc)

Multi‐layered fmhc were prepared using a layer‐by‐layer approach. For fixation of the free‐standing fiber‐meshes, Vaseline was applied as a sealant at the outer edges via extrusion from a syringe. The basal fiber mesh was cut from the frames using a scalpel and secured on a transparent plastic sheet using a Vaseline ring below and above, followed by application of 100 µL (18.8 µL cm^−^^2^) of the SSPS on top. Through repetition of the three steps (fiber mesh–Vaseline–SSPS) making up one layer, 5‐layer composite constructs were created. Subsequently, constructs were incubated in a humid chamber for self‐assembly of the SSPS to form hydrogel networks at 37°C for 7 days to ensure complete gelation of the low‐concentrated spider silk solution before further analysis. As controls without fibers, the protein solution was incubated identically for gelation. The Vaseline sealant ring was removed after gelation using a ring puncher and a scalpel.

### 4.4 Scanning Electron Microscopy (SEM)

For morphological characterization, hydrogels and fmhc were fixed with 2.5% glutaraldehyde in 80 mm HEPES, 3 mm CaCl_2_, pH 7.3 for 1 at RT. After washing twice in the same buffer without glutaraldehyde for 15 min, the samples were dehydrated in an ethanol series (30, 50, 70 and 80%) for 30 min each, then in 90 and 100% (v/v) ethanol (VWR, Darmstadt, Germany) for 1 h. Samples were stored in fresh 100% (v/v) ethanol at 4 °C, overnight and subsequently critical point dried using an automated CPD300 device (Leica, Wetzlar, Germany). The prepared samples and air‐dried fiber sheets were mounted to stubs using carbon tape and investigated using scanning electron microscopy after sputter coating with a 2 nm platinum layer (Sputter coater EM ACE600, Leica, Wetzlar, Germany). Images were recorded using a Thermo Scientific (FEI) Apreo VS with a Field Emission Gun at 2–3 kV and SE2 and BSE detectors. For fiber meshes, the mean fiber diameter was determined using ImageJ 1.53q (National Institutes of Health, Bethesda, MD, USA) to measure the diameters of 100 fibers per sample.

### 4.5 Secondary Structure Characterization Using FTIR

Secondary structures of as spun and post‐treated spider silk fiber meshes were analyzed using Fourier transform infrared (FTIR) spectroscopy (Tensor I with IR Microscope Hyperion 3000, Bruker, Billerica, MA, USA). For each spectrum, 100 absorbance scans with a resolution of 4 cm^−1^ in the range between 800 and 4000 cm^−1^ were accumulated and averaged. Fourier self‐deconvolution (FSD) and curve fitting of the amide I region (1604–1705 cm^−1^) was performed using the OPUS software (Bruker, Billerica, MA, USA), and the secondary structure elements were assigned according to Hu et al. [59].

### 4.6 Analysis of Form Stability

To visualize the enhanced form stability of fmhc, circular ring frames (inner diameters ranging from 7 mm to 15 mm) and a holder were designed using the software FreeCAD (version 0.19). The structures were printed using an Ultimaker 3 FDM (fused‐deposition‐modeling) printer and standard polylactic acid (PLA) filaments (Ultimaker, Zaltbommel, Netherlands). In order to decrease the hydrophobicity, the rings were coated with eADF4(C16) according to Borkner et al. [60]. Therefore, 20 mg mL^−1^ eADF4(C16) was dissolved in 6 m guanidinium thiocyanate and dialyzed against 20 mm ammonium bicarbonate buffer, pH 9, to generate a 10 mg mL^−1^ solution. 3D printed rings were treated with oxygen plasma (5 min, 100 W, 0.2 mbar) (MiniFlecto, plasma technology, Herrenberg‐Gültstein, Germany) and subsequently immersed in the eADF4(C16) solution for 2 min. After drying in air, coatings were rendered water stable by dipping into 70% (v/v) ethanol for 2 min, and then they were dried in air again. Hydrogels and fmhc were applied on top of the coated ring frames and tested for their ability to bridge the inner hole.

### 4.7 Rheological Characterization

Rheological measurements of fmhc constructs (*n* = 3) and eADF4(C16) hydrogels (*n* = 4) were performed using a Discovery Hybrid Rheometer 2 (TA Instruments, New Castle, DE, USA) controlled using the TRIOS software (version #5.1.1.46572, TA Instruments, New Castle, DE, USA) with a 25 mm sandblasted parallel plate‐plate geometry at a controlled temperature of 25 °C. To prevent drying effects, a wet sponge adapter was used. The measurement protocol included an initial frequency sweep at 0.1% strain to stay in the linear viscoelastic region (LVE) and an angular frequency of 0.1–100.0 rad s^−1^. After a soaking time of 10 s, an amplitude sweep was recorded at 10.0 rad s^−1^ and a strain of 0.01–1000.0%. The shear modulus *G* was extracted from the storage modulus of the initial frequency sweep. The Young's modulus *E* was estimated from the shear modulus *G* using the following equation [3, 53]:

$$E=2\;G\;\left(1+\nu \right)$$

with *ν* being the Poisson's ratio, estimated to a value of 0.5 under the assumption $G^{\prime }\gg G^{\prime \prime }$ for hydrogels [3].

### 4.8 Cell Cultivation

NIH/3T3 mouse embryonic fibroblasts expressing the FUCCI cell cycle reporter system were cultured in high‐glucose (4.5 g/L) Dulbecco's modified Eagle's medium (Gibco, ThermoFisher Scientific, Waltham, MA, USA) supplemented with 10% (v/v) bovine calf serum (Sigma‐Aldrich, Merck, Darmstadt, Germany), 100 U mL^−1^ penicillin/streptomycin (BioSell), 4 mm glutamine (Gibco, USA), 1 mm sodium pyruvate (Gibco, USA) at 37°C in a humidified incubator (95% relative humidity, 5% (v/v) CO_2_ (Heracell, ThermoFisher Scientific, Waltham, MA, USA). 3 µg mL^−1^ puromycin (Gibco, USA) ensured selection of transduced cells containing the plasmid encoding the FUCCI cell cycle reporter system. Non‐transduced NIH/3T3 cells were cultivated similarly without the presence of puromycin. Cell number and viability were determined using trypan blue (Sigma‐Aldrich, Germany) and an automated cell counter (BioRad, Hercules, CA, USA). Cells were split before reaching confluency using Trypsin/EDTA solution (0.05%/0.02% (w/v), Gibco, USA).

### 4.9 Preparation of Recombinant Spider Silk Hydrogels and Cell Encapsulation

eADF4(C16)‐RGD was solubilized and dialyzed as described above. After centrifugation and determination of concentration, the protein solution was concentrated via dialysis against 25% (w/v) polyethylene glycol (PEG, 40 000 g mol^−1^, Carl Roth GmbH & Co. KG, Karlsruhe, Germany) and centrifuged again at 17 000 x *g*, 4°C for 10 min to remove protein aggregates. 3.5% and 1.16% (w/v) (35 and 11.6 mg mL^−1^) spider silk protein solutions were pre‐gelled at 37°C and mixed with 15% (v/v) cell culture medium (see above) containing 2 × 10^6^ NIH/3T3 FUCCI reporter cells per ml to obtain a final gel concentration of 3% (w/v) and 1% (w/v), respectively. Cell‐loaded hydrogels were gelled in an overhead‐shaker (Elmi, Riga, Latvia) at 5 rpm at 37°C in a cell culture incubator overnight and transferred into 8‐well chamber slides (ibidi GmbH, Gräfelfing, Germany). 300 µL cell culture medium was added on top after one hour and was changed every second day.

### 4.10 Cell Compatibility of fmhc

Fiber meshes cut to a suitable size fitting in the chambers of 8‐well slides were fixed therein using a 5 µL drop of a 1% (w/v) eADF4(C16)‐RGD hydrogel precursor solution. 18.8 µL of the precursor solution were then applied on top of the fiber mesh for subsequent hydrogel self‐assembly at 37°Cin a humidified incubator. After sterilization for 30 min using ultraviolet (UV) irradiation, these monolayer fmhc and 1% eADF4(C16)‐RGD hydrogels were seeded with NIH/3T3 FUCCI cells at 5000 cells cm^−2^ in 300 µL cell medium and analyzed using confocal microscopy for evaluation of cell proliferation.

For analysis of cell morphology, non‐transduced NIH/3T3 cells were seeded on top of the matrices (50,000 cells cm^−2^) and after 24 h of cultivation washed using PBS and fixed using 3.7% (v/v) paraformaldehyde (PFA) in PBS (15 min, ambient conditions). After permeabilization using 0.1% (v/v) Triton X‐100 in PBS (15 min, ambient conditions), the cell nuclei and cytoskeleton were stained using 300 nm 4’,6‐diamidino‐2‐phenylindole dihydrochloride (DAPI, Sigma–Aldrich, Germany) and 200 nm phalloidin tetramethyl rhodamine B isothiocyanate (Phalloidin‐red, Sigma‐Aldrich, Germany) in PBS (60 min, RT, dark). Samples were washed one more time with PBS and imaged in fresh PBS.

### 4.11 Fluorescence and Confocal Laser Scanning Microscopy

Confocal images of cell‐loaded hydrogels were collected using an inverted TCS SP8 confocal laser scanning microscope (Leica, Wetzlar, Germany) equipped with a 10× objective and lasers using excitation wavelengths of 488 nm and 552 nm and the associated LAS X software version 3.5.7.23225. Sequential acquisition settings as well as optimized emission ranges of the HyD hybrid detector were used to avoid inter‐channel crosstalk. Z‐stack series over a distance of 100 µm with 10 µm increments were collected for 9 randomly chosen regions for each condition over a period of 7 days. Cells cultivated on top of the fabricated scaffolds were visualized using a DMi8 fluorescence microscope (Leica, Wetzlar, Germany) equipped with a 20× objective and DAPI/TXR filter sets.

### 4.12 Image Quantification

Fluorescence and confocal images were processed using ImageJ. For evaluation of cell proliferation, a maximum intensity projection was made over the z‐stacks, followed by channel merging. The color thresholding function was used to distinguish between red, yellow and green cell nuclei, and the analyze particles function was used for nuclei quantification in hydrogels. Due to background signals of fmhc, nuclei were counted manually.

To assess the cell morphology, a threshold was set for DAPI and phalloidin channels, followed by applying the measurement function to quantify the signal area. For relative quantification of the average spreading behavior, phalloidin signal area was normalized to DAPI signal area and compared between the tested conditions.

### 4.13 Statistical Analysis

Data were presented as mean ± standard deviation. Statistical analysis was conducted using the Origin software (version 9.6.0.172, OriginLab Corporation, MA, USA). One‐way ANOVA was used for means comparisons. A *p*‐value < 0.05 was considered statistically significant.

## Author Contributions

**C.H**.: data curation, formal analysis, investigation, methodology, validation, visualization, writing – original draft, review & editing. **T.S**.: conceptualization, funding acquisition, methodology, project administration, resources, supervision, validation, writing – review & editing.

## Conflicts of Interest

T.S. is co‐founder and shareholder of the company AMSilk GmbH.

## Supporting information




**Supporting file**: marc70059‐sup‐0001‐SuppMat.docx.

## Data Availability

The data that support the findings of this study are available from the corresponding author upon reasonable request.
