# Supplementary material for: Fiber Reinforcement of Soft Spider Silk Hydrogels
Source: Macromol Rapid Commun. 2025 Sep 16;47(7):e00475. doi: 10.1002/marc.202500475 (PMC13047472; doi:10.1002/marc.202500475)
Supplement: Supplementary file 1 — Supporting file: marc70059‐sup‐0001‐SuppMat.docx. [file MARC-47-e00475-s001.docx]

Supporting Information

Fiber-reinforcement of soft spider silk hydrogels

Christina Heinritz and Thomas Scheibel*


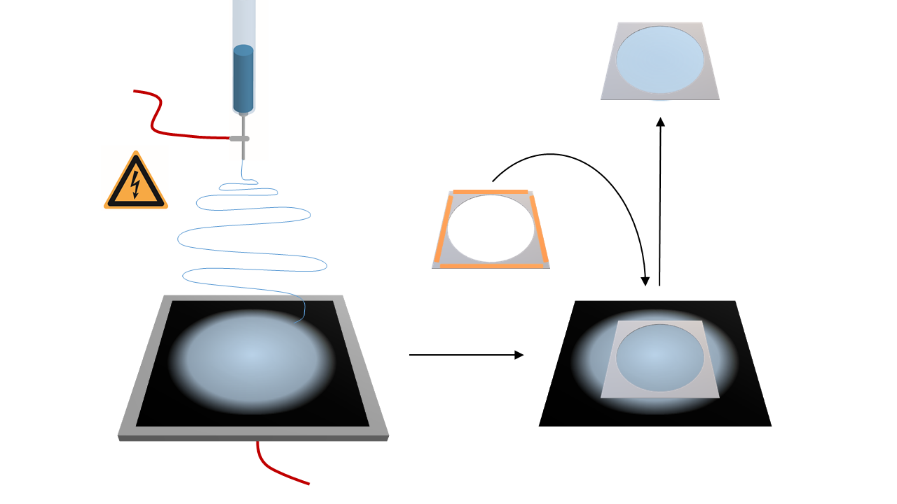


**Figure S1**: Schematic representation of the electrospinning process and subsequent transfer of nanofiber sheets onto custom-3D-printed frames to yield free-standing meshes.


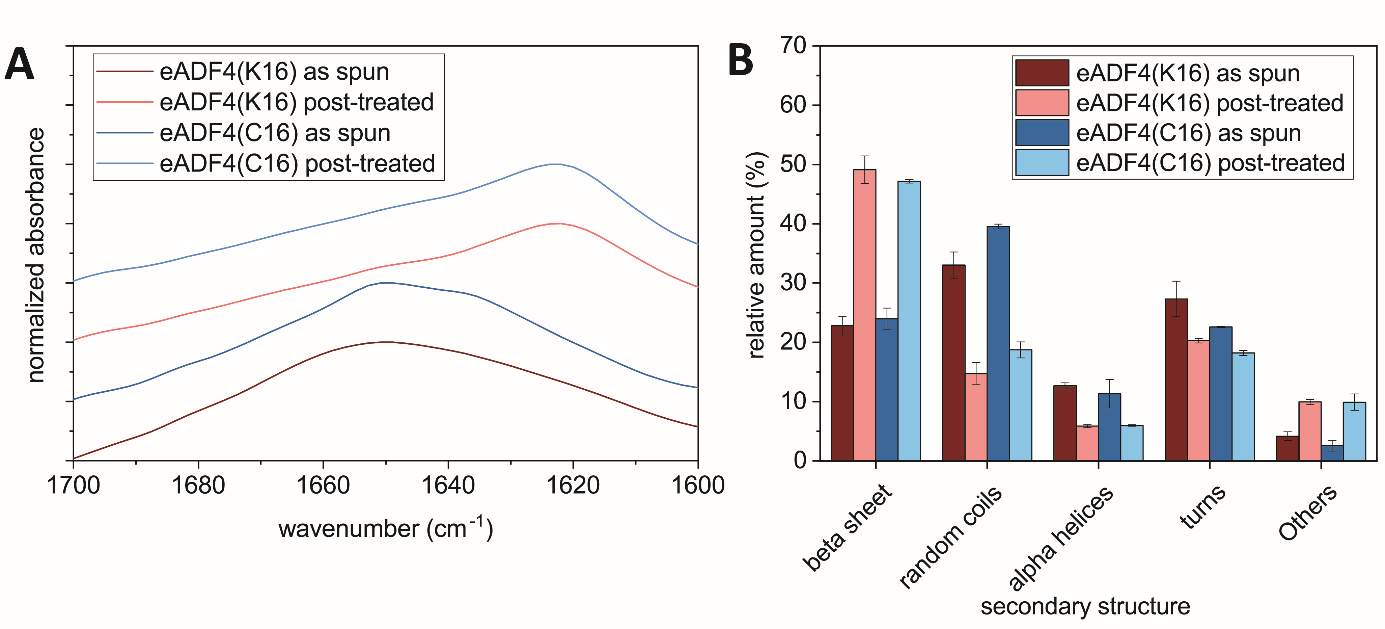


**Figure S2.** (A) Amide I region of representative normalized FTIR spectra of electrospun silk protein fiber meshes before (as spun) and after post-treatment with methanol. (B) Secondary structure elements as determined using Fourier self-deconvolution revealing an increased β-sheet content after post-treatment.


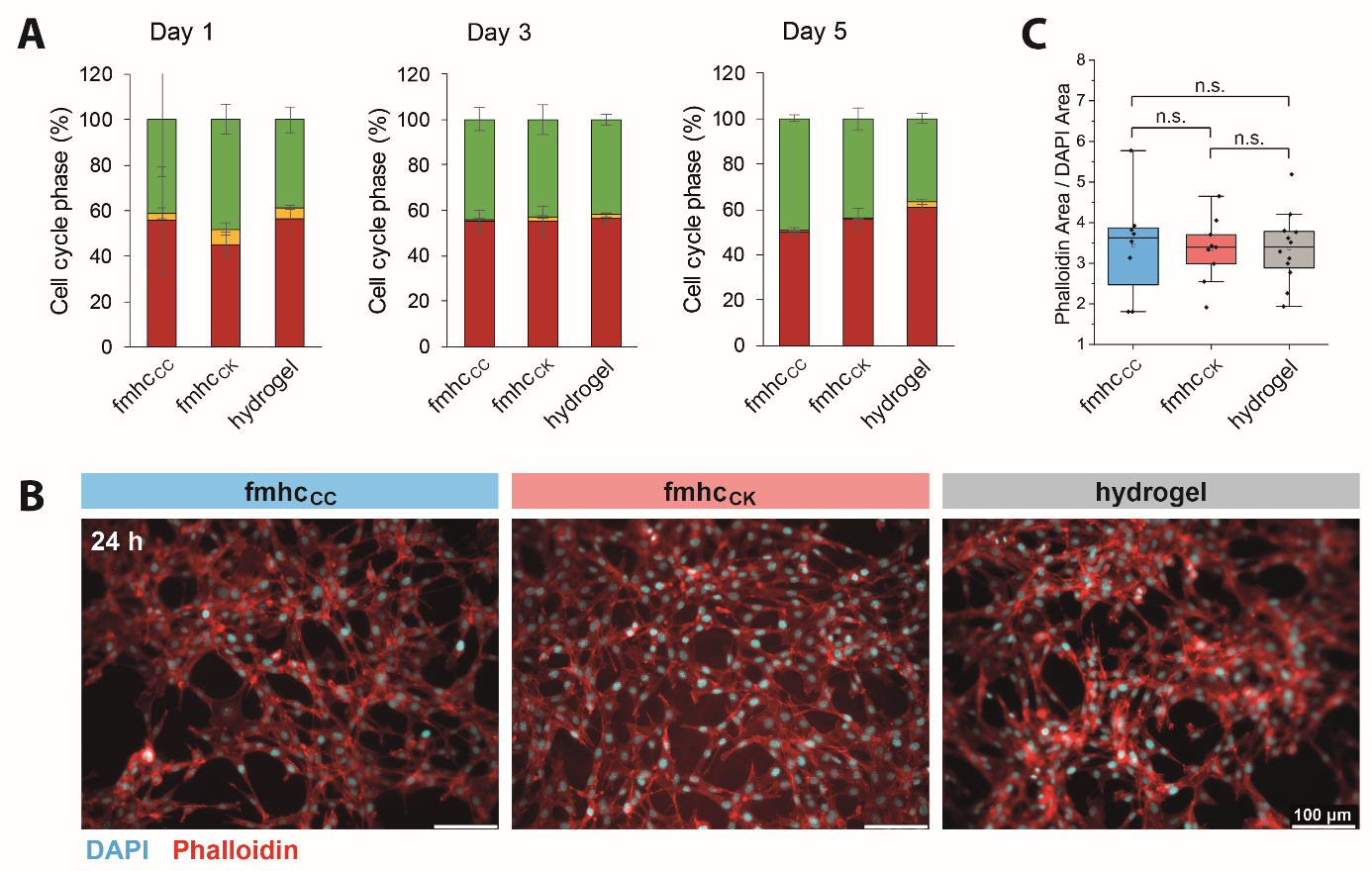


**Figure S3.** Cultivation of fibroblasts on top of monolayered fmhc and 1% control hydrogels. For the hydrogel fraction, the recombinant spider silk protein eADF4(C16)-RGD was used to provide attachment sites for the cells. (A) Assessment of NIH/3T3 proliferation-reporter cells in the proliferative state (S/G2/M phases, green, and G1/S transition phase, yellow) and in the G1 cell cycle arrest (red) at days 1, 3, and 5, respectively. Day 7 was removed from the analysis due to cell confluency. (B) NIH/3T3 cells fixed after 24 h and stained with DAPI (blue, nuclei) and phalloidin (red, actin cytoskeleton) exhibited a similar spread morphology for the three conditions. Scale bar = 100 µm. (C) Evaluation of average phalloidin signal per cell showed no significant differences.
